# Body size and trophic level increase with latitude, and decrease in the deep-sea and Antarctica, for marine fish species

**DOI:** 10.7717/peerj.15880

**Published:** 2023-09-07

**Authors:** Han-Yang Lin, Mark John Costello

**Affiliations:** 1 Institute of Marine Science, University of Auckland, Auckland, New Zealand; 2 Faculty of Biosciences and Aquaculture, Nord University, Bodo, Norway; 3 School of Environment, University of Auckland, Auckland, New Zealand

**Keywords:** Latitudinal gradient, Depth gradient, Fish, Trophic level, Body size, Depth zones

## Abstract

The functional traits of species depend both on species’ evolutionary characteristics and their local environmental conditions and opportunities. The temperature-size rule (TSR), gill-oxygen limitation theory (GOLT), and temperature constraint hypothesis (TCH) have been proposed to explain the gradients of body size and trophic level of marine species. However, how functional traits vary both with latitude and depth have not been quantified at a global scale for any marine taxon. We compared the latitudinal gradients of trophic level and maximum body size of 5,619 marine fish from modelled species ranges, based on (1) three body size ranges, <30, 30–100, and >100 cm, and (2) four trophic levels, <2.20, 2.20–2.80, 2.81–3.70, >3.70. These were parsed into 5° latitudinal intervals in four depth zones: whole water column, 0–200, 201–1,000, and 1,001–6,000 m. We described the relationship between latitudinal gradients of functional traits and salinity, sea surface and near seabed temperatures, and dissolved oxygen. We found mean body sizes and mean trophic levels of marine fish were smaller and lower in the warmer latitudes, and larger and higher respectively in the high latitudes except for the Southern Ocean (Antarctica). Fish species with trophic levels ≤2.80 were dominant in warmer and absent in colder environments. We attribute these differences in body size and trophic level between polar regions to the greater environmental heterogeneity of the Arctic compared to Antarctica. We suggest that fish species’ mean maximum body size declined with depth because of decreased dissolved oxygen. These results support the TSR, GOLT and TCH hypotheses respectively. Thus, at the global scale, temperature and oxygen are primary factors affecting marine fishes’ biogeography and biological traits.

## Introduction

Latitudinal diversity gradients (LDGs) integrate over the local and regional patterns where species have evolved and survived on both ecological and evolutionary time scales (Ekman, 1953; Gaston, 2000; Willig, Kaufman & Stevens, 2003; Pontarp et al., 2019). In contrast to expectations that species richness decreases from the equator to the poles, recent studies have shown that the LDG of marine taxa are bimodal with a dip at or near the equator (Powell, Beresford & Colaianne, 2012; Chaudhary, 2019; Chaudhary, Saeedi & Costello, 2016, 2017; Chaudhary & Costello, 2023; Arfianti & Costello, 2020; Lin et al., 2021). This was not the case during the last glacial maximum (Yasuhara et al., 2020), and dip has been deepening faster in concert with recent climate change (Chaudhary et al., 2021), indicating that the LDG in terms of species richness is related to temperature (Lin et al., 2021). However, how biological traits of species vary with latitude globally has not been studied. Functional traits characterize an organism’s phenotype, indicating how it may interact with the physical, chemical, and biological environments (Hooper et al., 2005). Body size, trophic level, and depth distribution are the most widely used numerical functional traits and have relatively complete records for many fish species (Costello et al., 2015; Froese & Pauly, 2019). Thus, in this study, the latitudinal and depth gradients of body size and trophic level of marine fish were studied.

A positive relationship between body size and latitude has been demonstrated in freshwater and marine ectotherms (Lindsey, 1966; Fisher, Frank & Leggett, 2010; Bartels et al., 2020). The phenomenon where populations within a species, and/or species, have a smaller size in low latitudes (warm environment) and a larger size in high latitudes (cold environment) is known as the temperature-size rule (TSR) (Atkinson, 1994). In the marine environment, dissolved oxygen is another factor that limits marine organisms’ body size (Forster, Hirst & Atkinson, 2012; Hoefnagel & Verberk, 2015). This phenomenon for fish has been explained by the gill-oxygen limitation theory (GOLT) that indicates the oxygen supply depends on the gill surface area related to the body mass volume (Pauly & Cheung, 2018). The ratio of gill surface area to body mass decreases when individuals grow. Therefore, an organism’s body size is limited by the oxygen needed for maintaining its metabolic demands, whereby oxygen concentration in water declines with warming and metabolic demand increases (Pauly & Cheung, 2018). Thus, GOLT provides a mechanism to explain the TSR.

More marine herbivorous or omnivorous fish exist in the low than high latitudes, and few herbivores in areas with an annual average temperature of below 20 °C (Floeter et al., 2005; Behrens & Lafferty, 2007; González-Bergonzoni et al., 2012; Dantas et al., 2019). This may be explained by the temperature constraint hypothesis (TCH) which states that low temperatures constrain the efficiency of digestion for marine ectothermic herbivores (Gaines & Lubchenco, 1982). However, a metanalysis and study on a temperate reef fish species concluded the TCH did not explain herbivory gradients with latitude but provided no alternative hypothesis (Johnson et al. 2020; Knight, Guichard & Altieri, 2021).

Therefore, the hypotheses TSR and TCH directly, and GOLT indirectly, suggest that temperature is the primary driver to constrain the body size and thereby influence the trophic level of fish, as supported for the effect of paleoclimate on body size evolution of fishes (Troyer et al., 2022). In this study, we correlate functional traits with temperature and dissolved oxygen, and assess whether the relationships support TSR, GOLT, and TCH hypotheses. In addition, we included salinity as a comparative variable because it is a widely used indicator of oceanographic water masses and associated variability of water masses in an area.

Another environmental gradient of interest regarding the diversity pattern is depth. The deep sea below 200 m depth is dark, cold, and with low-dissolved-oxygen across all latitudes (Costello & Breyer, 2017; Sayre et al., 2017; Basher & Costello, 2020). Therefore, it would be expected that the LDG may vary in the surface depth zone but be relatively constant in the deep sea. Previous studies found that relatively more species of fish with larger body size and higher trophic level occurred with greater depth (Smith & Brown, 2002; Lamprakis, Kallianiotis & Stergiou, 2008; Mindel et al., 2016). However, these studies focused on specific fish groups and study areas. Placing these patterns on a global scale will provide a more robust and more general theoretical understanding of variation. Besides, how body sizes and trophic levels of marine fish change among latitudes and with depth at the global scale have not been studied previously.

This study describes the gradients of body size and trophic level of marine fish among latitudes in different depth zones, and along depth zones with latitude, globally. The relationship between functional traits and environmental variables is presented. We hypothesize that fishes with smaller body size and lower trophic levels dominate warmer waters (low latitudes, shallow depths), and the reverse in cooler waters (high latitudes, deep depths).

## Materials and Methods

### Species data

To avoid problems with spatially biased sampling, we used species ranges as is conventional in biogeography. The modelled geographic distribution, including latitude and depth, of the 5,619 fish species with available ranges was obtained from AquaMaps (Kaschner et al., 2019) and represents about one-third of all marine fish species (Froese & Pauly, 2019). A full list of the species is in Supplementary Information at Figshare (https://doi.org/10.6084/m9.figshare.19314317.v3). AquaMaps models field observations with environmental variables to predict the environmental niche and thus geographic ranges of species (Kaschner et al., 2019). A species will only occur within its range where local environmental and ecological conditions are suitable, and its abundance will vary within its range in space and time. Thus, a species may not occur everywhere within its mapped range, and if its range changes over time, such as due to climate change, it may no longer occur within part of its mapped range. To prevent a species range extending into areas that are environmentally suitable but where it does not occur for evolutionary reasons (*e.g*., in a different ocean), AquaMaps limits species ranges to their known occurrence in FAO regions (Kaschner et al., 2019). The probability threshold of 0.0 (possibly present) was used as a conservative approach to ensure some species occurrences in most geographic cells following Lin et al. (2021).

### Species traits

Two functional traits, maximum body size as standard length in cm and trophic level, were retrieved from FishBase for each of the fish species (Froese & Pauly, 2019). Species of body size smaller than 30 cm (49% of species) and between 30 and 100 cm (40% of species) dominated in this dataset. Only 11% of species had body size larger than 100 cm in this dataset (Table 1, Fig. S1). These body size groups were only used for the purpose of illustrating patterns and were not used in the calculation of means. As the number of species of all marine fish from FishBase (*n* = 15,195) in these size categories was 62%, 32% and 6% respectively, our data were more inclusive of larger fish species (Table S1).

**Table 1:** Number of fish species in the three body size and four trophic level groups in the depth zones.

	Depth zone			
Fish group	Whole water column	0–200 m	201–1,000 m	1,001–6,000 m
All fish	5,619	4,102	1,184	333
**Body size**				
<30 cm	2,743	2,066	523	154
30–100 cm	2,260	1,618	497	145
>100 cm	616	418	164	34
**Trophic level**				
Herbivores and detritivores	242	241	1	0
Low-trophic-level omnivores	286	283	3	0
High-trophic-level omnivores	3,234	2,374	649	211
Carnivores	1,857	1,204	531	122

The trophic level index categorises adults of species according to their position in the food web, being <2.20 for herbivores and detritivores; 2.20–2.80 for omnivores with a preference for vegetable matter but also feeding on other prey (*e.g*., sponges, isopods, amphipods) (low-trophic-level omnivores); 2.81–3.70 for omnivores with a preference for animals but feeding on diverse prey (*e.g*., algae, bivalves, isopods, fish larvae) (high-trophic-level omnivores); and >3.70 for piscivores and carnivores with a preference for large decapods, cephalopods and fish (carnivores) (Stergiou & Karpouzi, 2002; Froese & Pauly, 2019). The proportions of the four trophic levels of all marine fish from FishBase (3%, 4%, 67%, 26%) were very similar to those in AquaMaps (4%, 5%, 58%, 33%), and thus our dataset is representative of trophic levels of all marine fish species (Table S1).

### Data analysis

The mean and standard error of body size and trophic level for all fish was calculated in 5° latitude bands between 75°S and 75°N and four depth zones: whole water column, surface (0–200 m), middle (201–1,000 m), and deep (1,001–6,000 m); reflecting the photic, mesophotic and aphotic zones of light penetration respectively (Costello & Breyer, 2017). This study also calculated the mean and standard error of body size and trophic level for all fish in 100 m depth bands from 0 to 3,500 m. Numbers of species, means and standard errors for traits groups in latitude and depth bands are shown as Tables S2–S8.

The mean body size and trophic level were correlated with the long-term decadal averages of monthly salinity (practical salinity unit, psu), and sea surface and bottom temperature (SST, SBT respectively) and dissolved oxygen (SDO, BDO respectively) in 5-degree latitude bands. The environmental variables were obtained from the Global Marine Environmental Datasets (Basher & Costello, 2020). Raster data of environmental variables were calculated as the mean value in every 5-degree latitude band between 75°S and 70°N. Generalized Additive Models (GAM) (Hastie & Tibshirani, 1990) were used to assess the relationship between latitudinal mean body size, trophic level and environmental variables in the same 5-degree latitude bands. A GAM is a nonparametric regression method that uses smooth functions of the predictors. Also, a GAM is flexible regarding the assumptions concerning the underlying statistical distribution of the data (Swartzman, Silverman & Williamson, 1995). The package “mgcv” (Wood, 2011) in R (R Core Team, 2018) was used for the GAM analysis. A GAM with Gaussian error distribution and identity link function was used for modelling. Smoothness selection(s) of thin plate regression splines was used for the model fitting process. The model was as follows:

${\rm Body\; size \;or \;Trophic \;level \sim s\; (each \;environmental \;variable)}$

Standard diagnostics including Quantile-Quantile (Q-Q) plot, minimized Generalized Cross-Validation (GCV) scores, maximized deviance explained and maximized adjusted r^2^, were used to assess distributional and smoothing assumptions (Wood, 2006). The deviance explained, adjusted r^2^, and GCV scores were recorded. The relationships of mean trophic level and body size to each environmental variable were plotted.

## Results

### Latitudinal gradients

**Figure 1:**
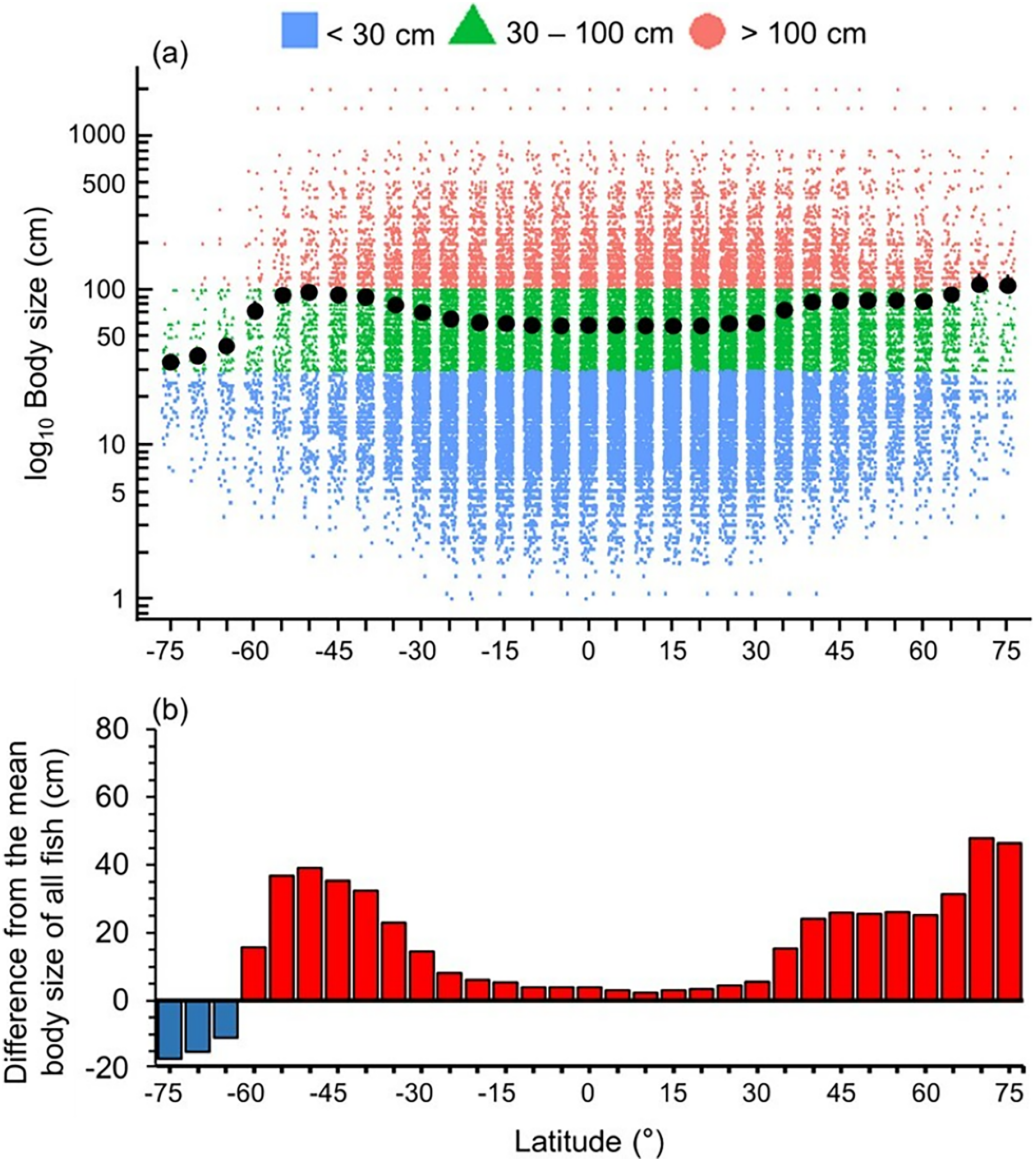
Latitudinal gradient of body sizes. Gradients of (A) mean body size with standard error (black dot and bar), and dot-plot of log_10_ maximum body size for <30 cm (blue squares), 30–100 cm (green triangles), and >100 cm (orange circles), and (B) difference from the mean body size of all fish in this dataset (50.7 cm) in 5-degree latitude bands in the whole water column. Each dot indicates one species.

Mean maximum body size of fish species was smaller in the Southern Ocean, tropics and sub-tropics (30°S and 30°N) (Fig. 1). Thus, there were relatively more large fish species in the temperate latitudes and Arctic, with the highest values at 50°S and 70°N (Fig. 1).

**Figure 2:**
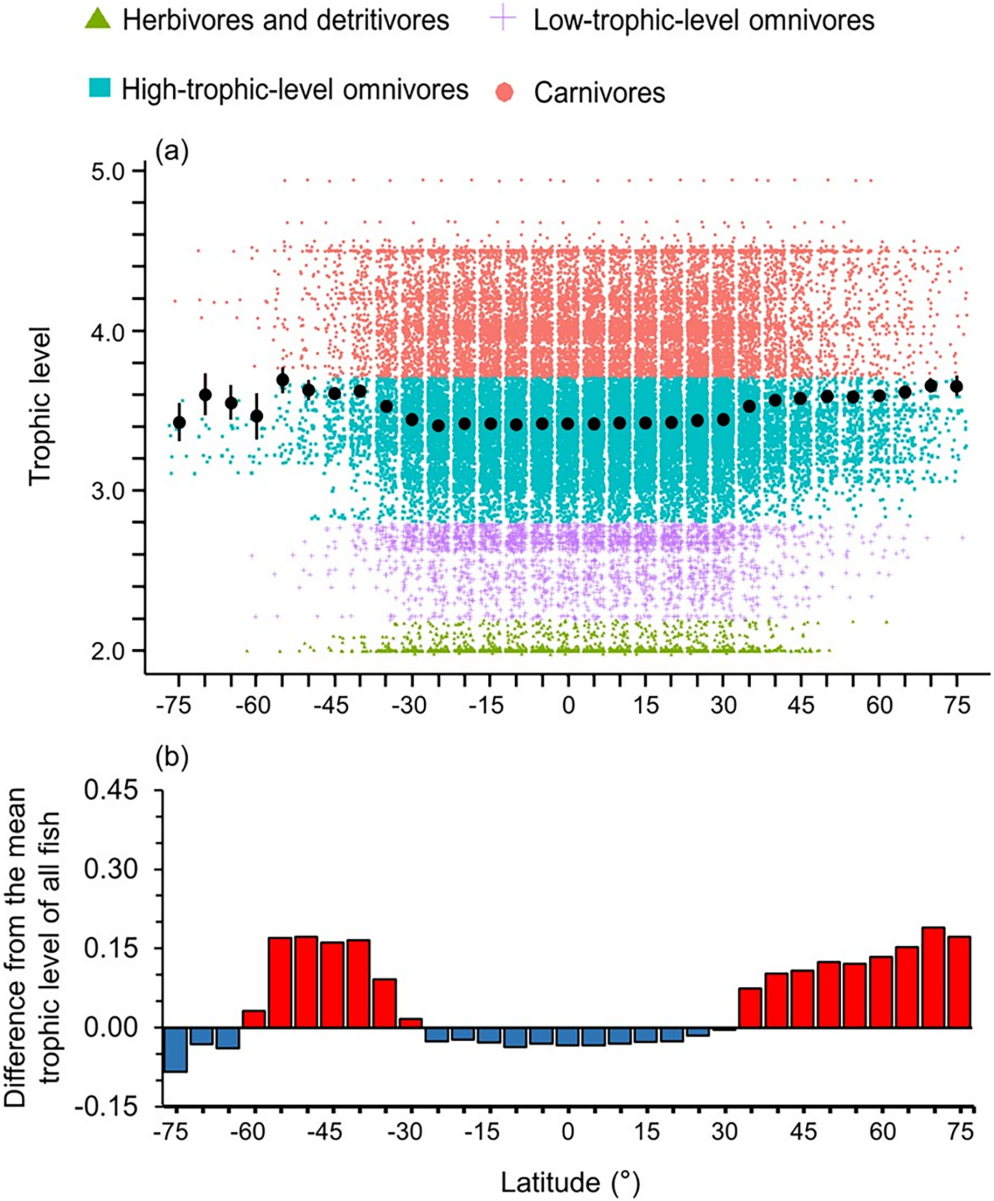
Latitudinal gradient of trophic levels. Gradients of (A) mean trophic level with standard error (black dot and bar), and dot-plot of trophic level among herbivores and detritivores (green triangles), low-trophic-level omnivores (purple crosses), high-trophic-level omnivores (teal squares), and carnivores (red circles); and (B) difference from the mean trophic level of all fish in this dataset (3.50) in 5-degree latitude bands in the whole water column. Each dot indicates one species.

High-trophic-level omnivore and carnivore species occurred across all latitudes (Fig. 2A). In contrast, herbivores and detritivores and low-trophic-level omnivores were absent in both poles, except for a few low-trophic-level omnivores distributed in the Arctic Ocean (Fig. 2A). Therefore, the mean trophic level and difference from the mean trophic level of all fish were lower in the tropics (30°S to 30°N) and the Southern Ocean (Fig. 2).

Overall, we found that the diversity of body sizes and trophic levels were highest in the tropics and subtropics, but the mean body sizes and trophic levels in this area were smaller and lower (Figs. 1 and 2). This is because there were more small, herbivorous and detritivorous fish species between 30°S and 30°N (Figs. 1 and 2).

**Figure 3:**
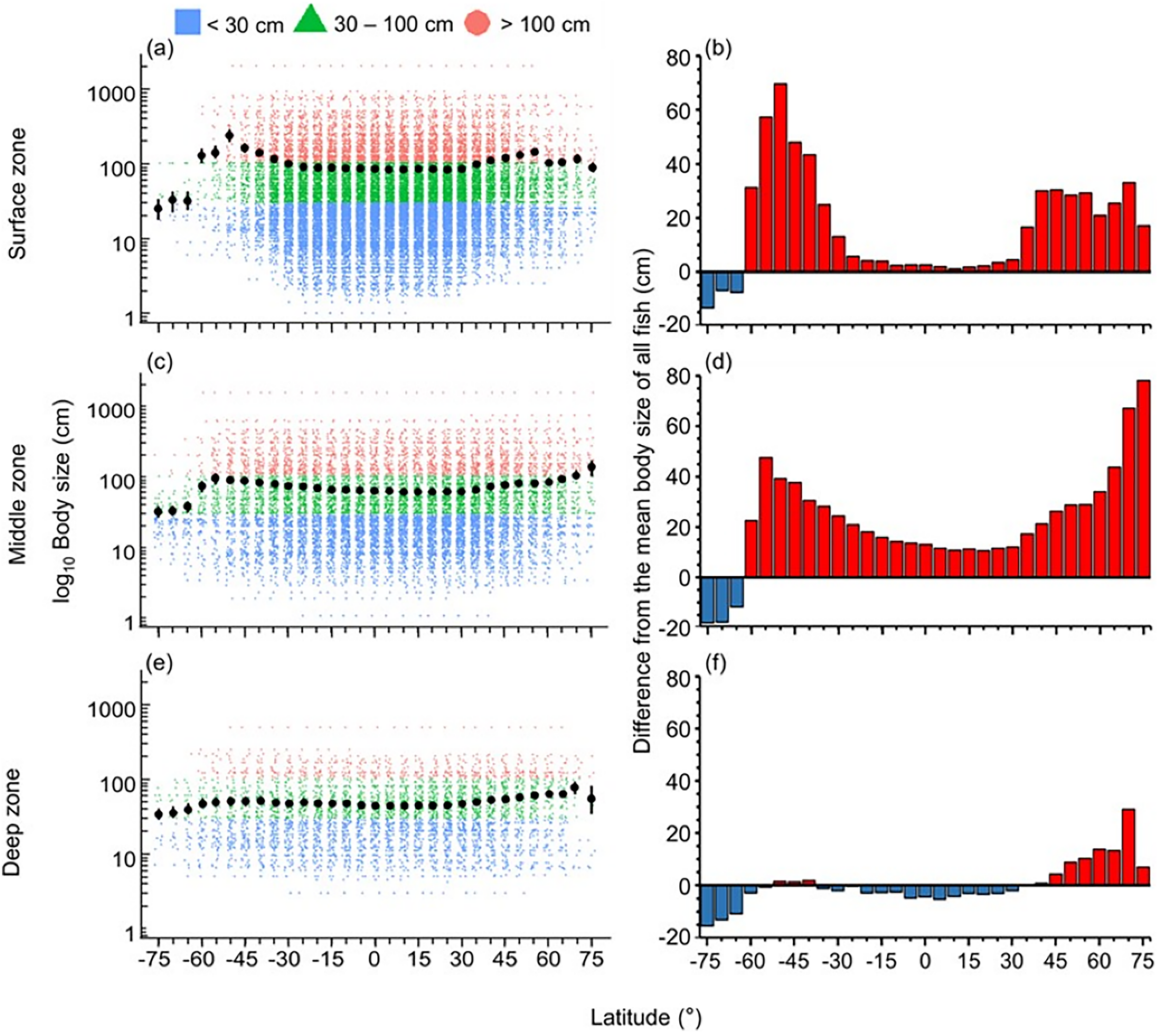
Latitudinal gradients of body sizes in depth zones. Gradients of (A, C, and E) mean body size with standard error (black dot and bar), and dot-plot of log_10_ maximum body size for <30 cm (blue squares), 30–100 cm (green triangles), and >100 cm (orange circles) fish, and (B, D, and F) difference from the mean body size of all fish in this dataset (50.7 cm) in 5-degree latitude bands in the surface (0–200 m), middle (201–1,000 m), and deep (1,001–6,000 m) zone . Each dot indicates one species.

**Figure 4:**
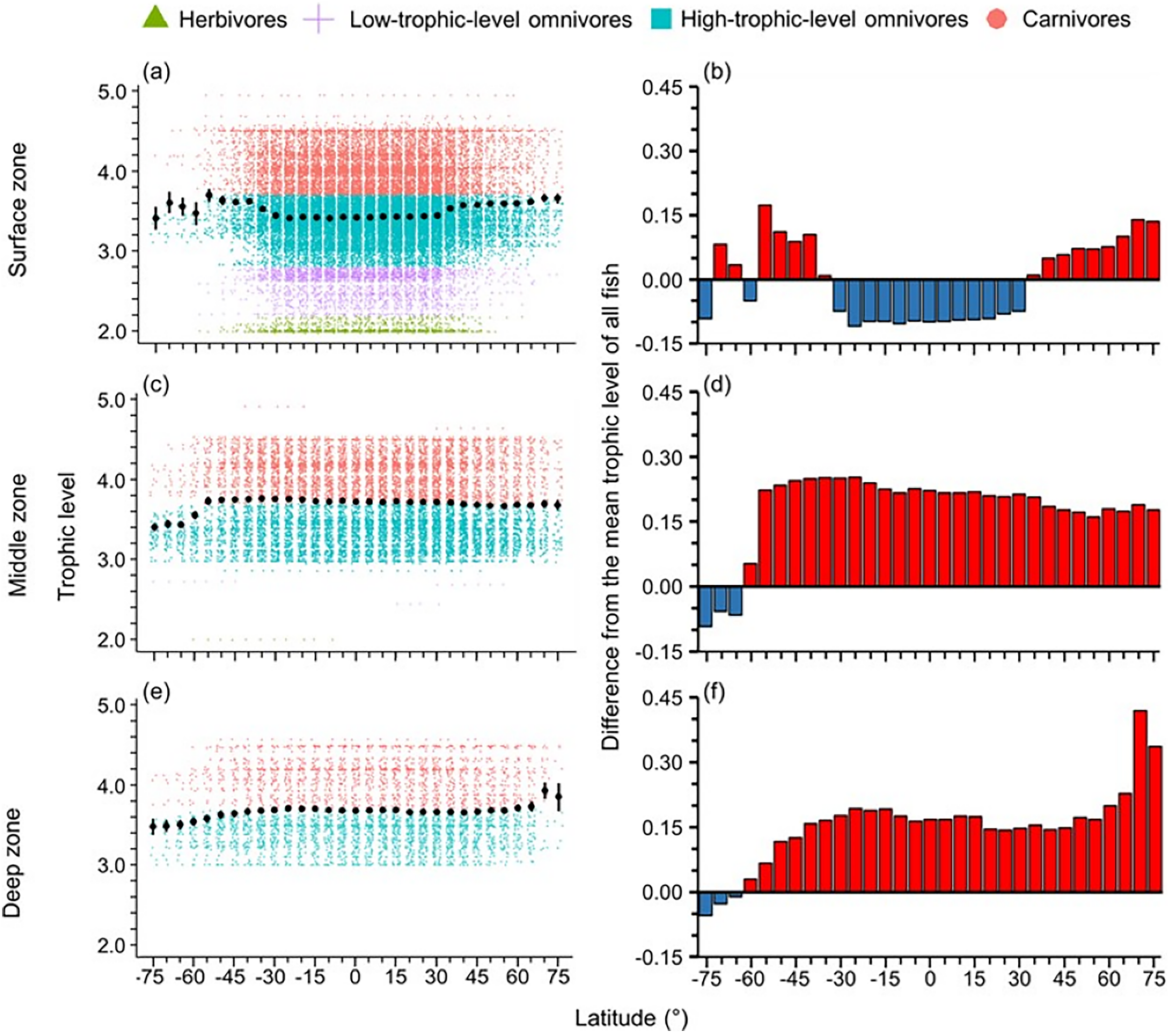
Latitudinal gradients of trophic levels in depth zones. Gradients of (A, C, and E) mean trophic level with standard error (black dot and bar), and dot-plot of trophic level among herbivores and detritivores (green triangles), low-trophic-level omnivores (purple cross), high-trophic-level omnivores (teal squares), and carnivores (orange circle) fish, and (B, D, and F) difference from the mean trophic level of all fish in this dataset (3.50) in 5-degree latitude bands in the surface (0–200 m), middle (201–1,000 m), and deep (1,001–6,000 m) zone. Each dot indicates one species.

The distribution of body size and trophic groups in the surface zone were similar to the pattern described in the whole water column because over 73% of marine fish occurred shallower than 200 m depth (Figs. 3 and 4). Compared to this near surface depth zone, fish were generally larger in the middle zone, and below average size in the deepest zone (Fig. 3). However, in all depth zones, the fish of the Southern Ocean were below average in body size and trophic level (Figs. 3 and 4). In contrast, southern temperate (40°S–50°S), northern temperate and Arctic fish (40°N–75°N) were above average in body size and trophic level in all depth zones (Figs. 3 and 4).

### Depth gradients

**Figure 5:**
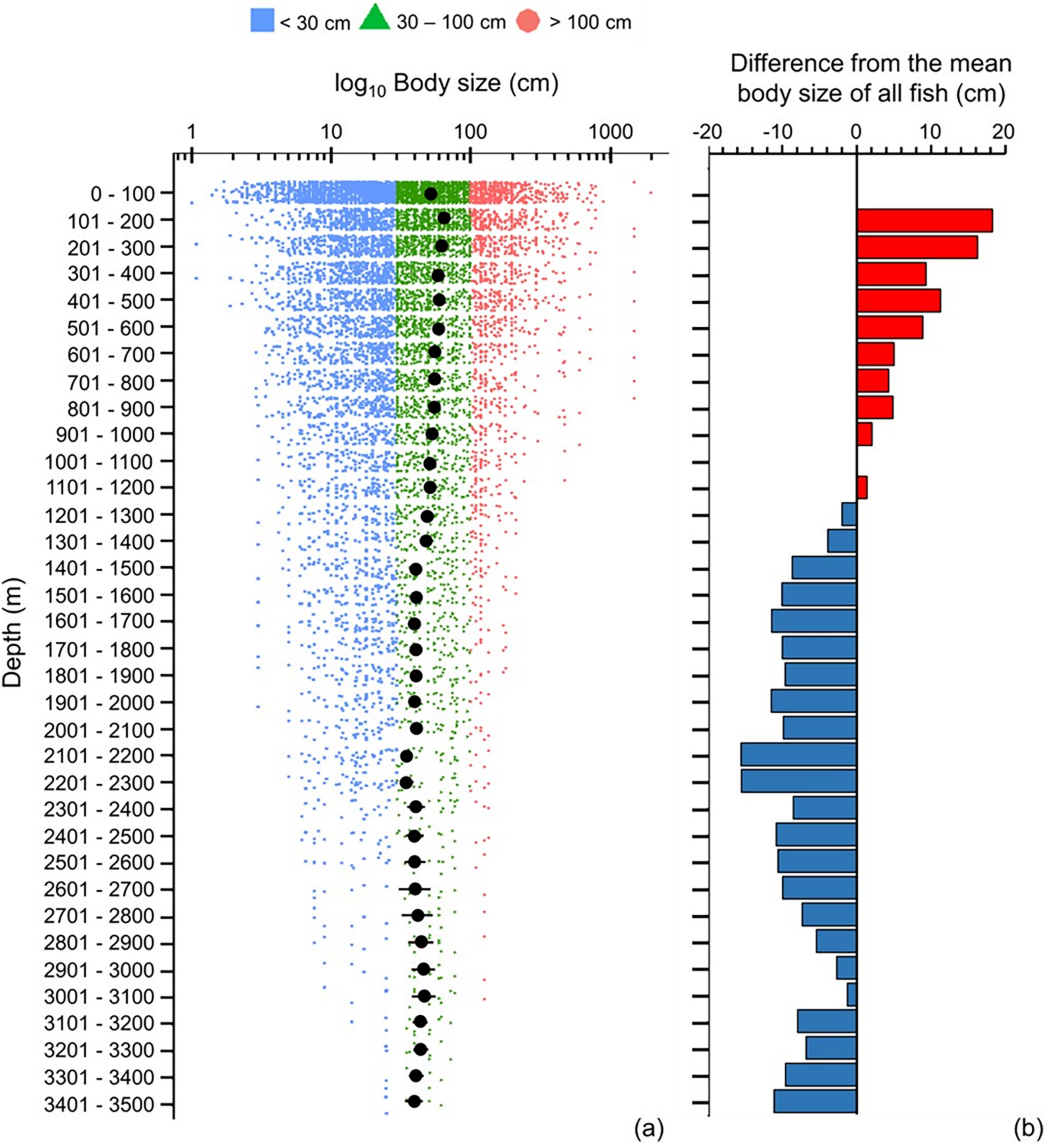
Depth gradient of mean body sizes. Depth gradients of (A) mean body size with standard error (black dot and bar), and dot-plot of log_10_ maximum body size for <30 cm (blue squares), 30–100 cm (green triangles), and >100 cm (orange circles), and (B) difference from the mean body size (50.7 cm) of all fish in this dataset along 100 m depth bands. Each dot indicates one species.

Across 100 m depth bands, mean maximum body size decreased with depth, and below 1,200 m fish species were below average in size (Fig. 5). However, the smallest and largest fish species occurred near the sea surface (<100 m), and they did not exist deeper than >500 and >900 m, respectively (Fig. 5A). Thus, with greater depth, fish’s body sizes converged closer to the average size (Fig. 5A).

**Figure 6:**
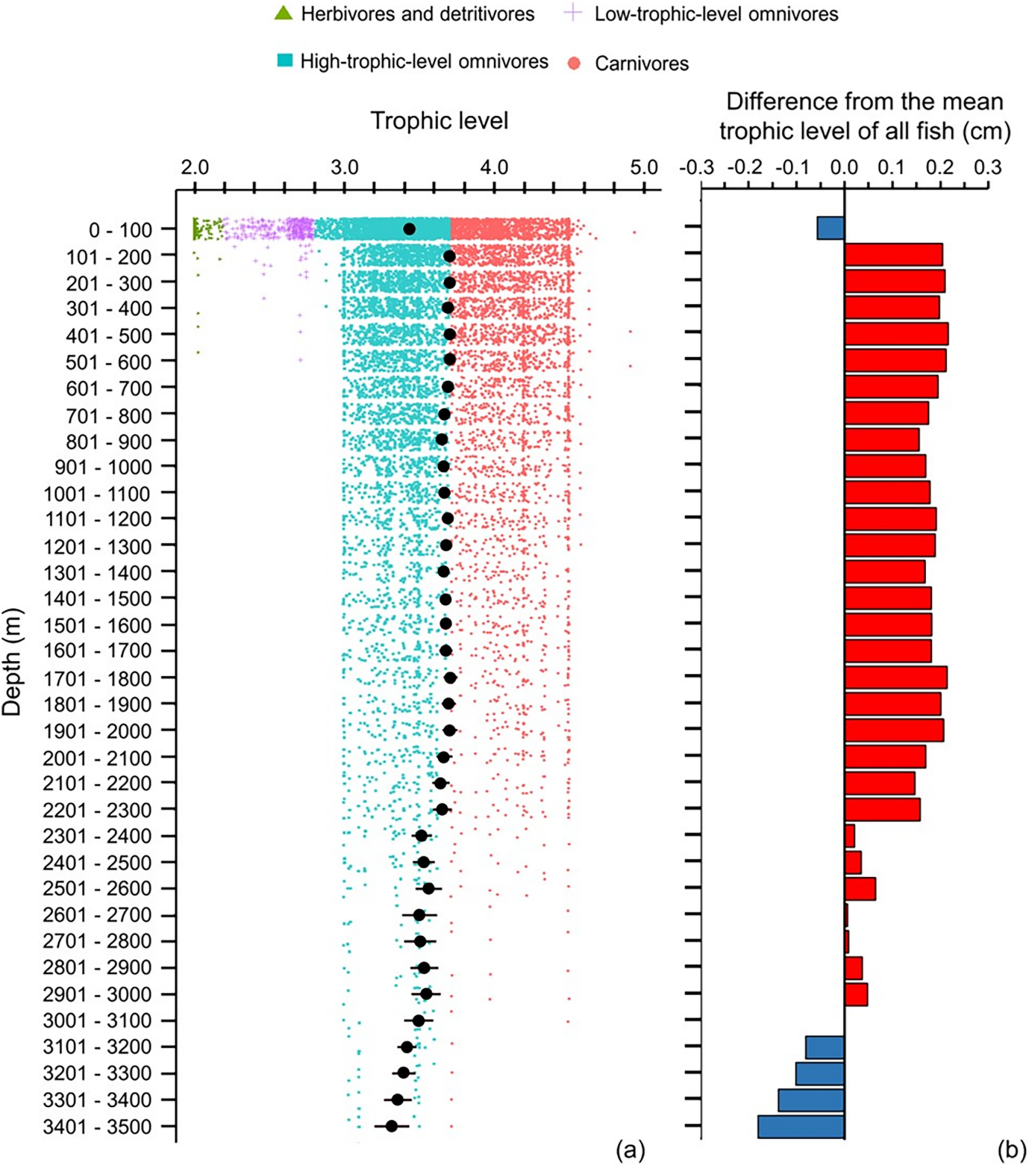
Depth gradient of mean trophic levels. Gradients of (A) mean trophic level with standard error (black dot and bar), and dot-plot of trophic level among herbivores and detritivores (green triangles), low-trophic-level omnivores (purple cross), high-trophic-level omnivores (teal squares), and carnivores (orange circle) fish, and (B) difference from the mean trophic level of all fish in this dataset (3.50) along 100 m depth bands. Each dot indicates one species.

The depth ranges of almost all (99%) of herbivores and detritivores, and 98% of low-trophic-level omnivores, included the surface zone (Fig. 6A). Only one species of herbivore and detritivore and three species of low-trophic-level omnivores could be found between 101 and 600 m (Fig. 6A). Deeper than 200 m, trophic level remained around 3.6–3.7 until a depth of 2,300 m (Fig. 6). When deeper than 2,300 m, trophic level decreased with depth and the standard errors got wider (Fig. 6). There were several subtle change points in depth trends of species’ maximum body size and trophic level at 100, 500, 900, 1,400, 1,800, 2,300, and 3,000 m, respectively (Figs. 5A and 6A).

### Environmental relationships

#### Environmental gradients

**Figure 7:**
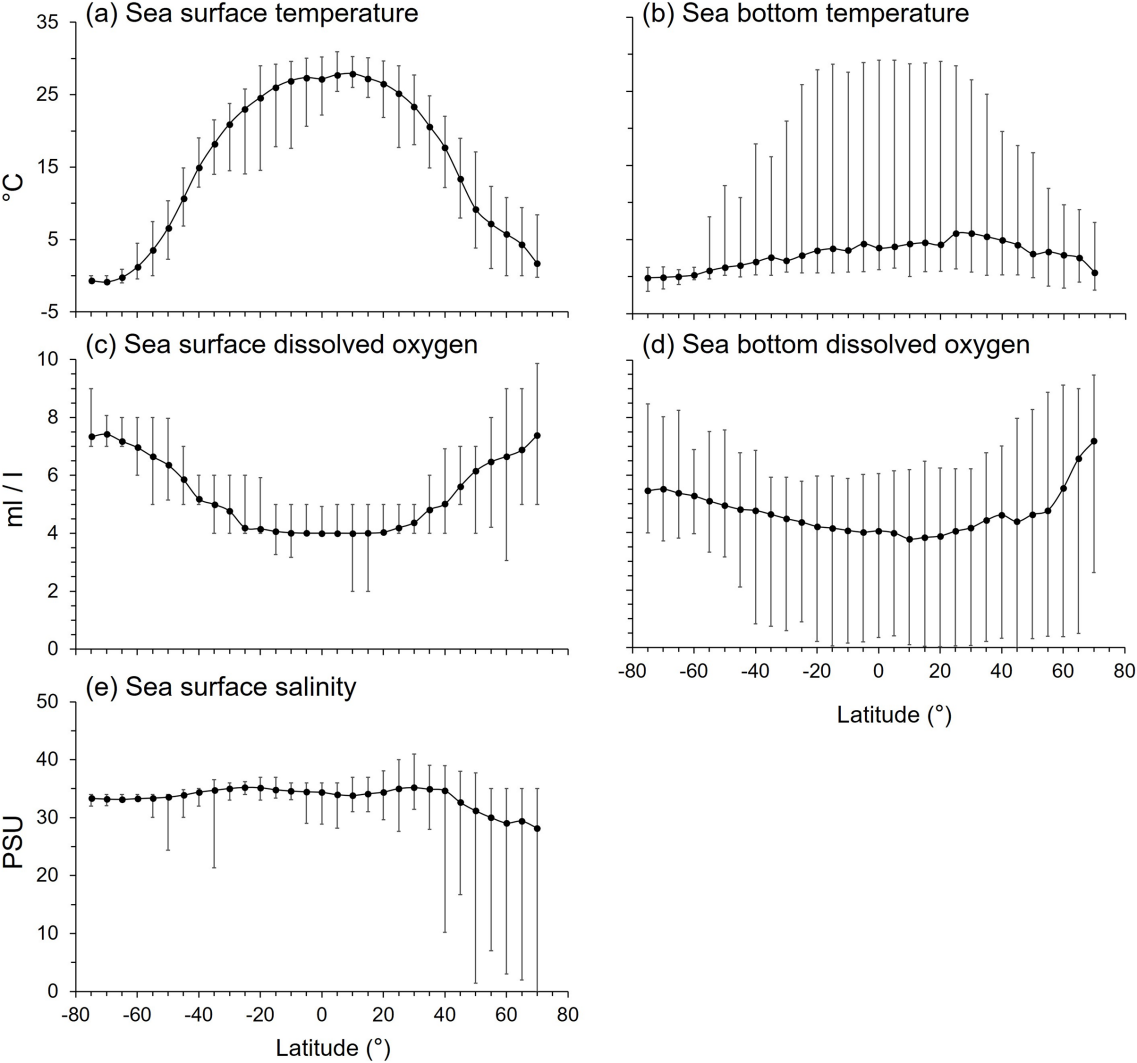
The latitudinal gradients of environmental variables. The latitudinal gradients of means (solid circle), maxima (top lines), and minima (bottom lines) of (A) sea surface temperature (°C), (B) sea bottom temperature (°C), (C) sea surface dissolved oxygen (ml/l), (D) sea bottom dissolved oxygen (ml/l), and (E) sea surface salinity (practical salinity unit, psu).

Mean sea surface temperature (SST) declined from the tropics to the poles with peaks at 10°N and 5°S (Fig. 7A). The narrower SST range near the equator and in the Southern Ocean indicated more stable temperatures (Fig. 7A).

The latitudinal gradient of mean sea bottom temperature (SBT) contrasted with SST because mean SBT was less than 6°C in all latitudes because of the dominating effect of the large area of deep sea in each latitude. SBT ranges were widest in the tropics to subtropics, and narrower at high latitudes because SBT varied more from shallow to deep sea in the tropical areas (Fig. 7B).

Mean sea surface dissolved oxygen (SDO) decreased from the poles to the tropics (Fig. 7C), being lowest between 25°N and 25°S. The range of SDO was widest in the Arctic Ocean (Fig. 7C). Mean sea bottom dissolved oxygen (BDO) was lowest at 10°N, then increased with southern and northern latitudes. The BDO range was wide in every latitude, especially in the northern temperate and Arctic Ocean (Fig. 7D).

Mean sea surface salinity varied between 28 to 35 psu with latitude, which is within the tolerance of marine organisms (Fig. 7E). However, mean sea surface salinity was lower and more variable north of 40°N (Fig. 7E). Thus, all variables studied here except BDO, namely SST, SBT, SDO and salinity, were at least five times more variable in the Arctic than Antarctic seas (Fig. 7).

#### Correlations

All environmental variables were significantly correlated with gradients in maximum body size and trophic level (Table 2). Mean SST was the primary factor that influenced the latitudinal gradients of both mean body size and trophic level. Fish with smaller body size and lower trophic levels were in the tropical latitudes with mean SST > 25 °C (Figs. 1–4, S2). Similarly, mean body size and mean trophic level decreased with warmer mean SBT (Fig. S2).

**Table 2:** Generalized additive model results for the latitudinal gradients of mean body size and trophic level of all fish against five environmental variables, ranked by deviance explained.

Environmental variable	Adjusted r^2^	Deviance explained (%)	Generalised cross-validation score	Degrees of freedom	*P*-value
**Body size**					
Sea surface temperature	0.88	91	46.71	7.86	***
Sea bottom temperature	0.74	80	94.77	6.71	***
Sea bottom dissolved oxygen	0.72	80	104.76	7.84	***
Sea surface dissolved oxygen	0.45	50	165.93	2.60	***
Sea surface salinity	0.19	22	230.22	1.00	**
**Trophic level**					
Sea surface temperature	0.76	79	0.002	4.07	***
Sea bottom dissolved oxygen	0.69	75	0.003	5.69	***
Sea surface dissolved oxygen	0.70	73	0.002	2.48	***
Sea bottom temperature	0.36	45	0.006	4.18	*
Sea surface salinity	0.36	39	0.005	1.39	**

**Notes:**

* *P*-value < 0.05.

** *P*-value < 0.01.

*** *P*-value < 0.001.

Mean SDO was 6 ml/l and mean BDO was 5 ml/l (Fig. S2). As with body size, mean trophic levels increased in latitudes with higher mean SDO and BDO. Mean trophic level was highest when mean SDO was 6.5 ml/l and BDO was 5 ml/l (Fig. S2).

Mean sea surface salinity was the least influential factor compared to temperature and dissolved oxygen. Most species, regardless of body size and trophic level, were living in salinities between 33 and 35 psu. Only a few species with larger body size and higher trophic level live in lower salinity water (Fig. S2).

## Discussion

### Latitudinal gradients

Our results showed that the diversity of fish species’ body size and trophic level were highest in the tropics and subtropics (between 30°S and 30°N). This may be because the tropics and subtropics also have a high diversity of associated predator, prey and competitor richness and habitats (Brown, 2014; Costello et al., 2015; Costello & Chaudhary, 2017). The warm temperature in the tropics, results in shorter generation times, higher rates of metabolism, faster rates of mutation, and faster selection, which generate and maintain higher biodiversity (Rohde, 1992; Wright et al., 2011; Brown, 2014). In addition, the tropics and subtropics have higher habitat complexity, notably coral reefs which may contain 27% of marine fish species (Costello, 2015; Froese & Pauly, 2019), and provide more ecological niches leading to higher species diversity (Rocha et al., 2005; Grosberg, Vermeij & Wainwright, 2012; Kovalenko, Thomaz & Warfe, 2012), and thus leading to higher traits diversity. Together these reasons allow species and trait diversity to originate and accumulate in the tropics and subtropics.

Our finding of a paucity of herbivores outside the tropics and subtropics supports the Temperature Constraint Hypothesis (TCH) that posits that the efficiency of digestion for plant materials is compromised in cooler environments (Gaines & Lubchenco, 1982). However, the mechanism behind this remains unclear (Johnson et al. 2020; Knight, Guichard & Altieri, 2021) and correlation does not imply causation.

In the surface zone, the latitudinal mean body size and mean trophic level were all lower in the tropics and subtropics but higher in the high latitudes except for the Southern Ocean. In contrast, neither varied significantly with latitude in the deep zone (*i.e*., deeper than 200 m) (Figs. 3 and 4). This was because there were more small (<30 cm) and lower trophic level (<2.80) fish species in the tropics and subtropics than in high latitudes and deep sea. Also, the diversity of body sizes and trophic level was similar across latitudes in the deep sea. These results support the initial expectation that latitudinal gradients of traits changed with latitude in the shallow depth zone but not in the deep sea because the deep sea has a more homogeneous environment with no light, low temperature, and low dissolved oxygen across latitudes (Costello & Breyer, 2017; Sayre et al., 2017; Basher & Costello, 2020).

### Depth gradients

Across 100 m depth bands, body size may be expected to be larger and the trophic level higher in the deep sea because the temperature is lower in the deep than shallow depths and food supply is largely dependent on secondary production in shallower waters. However, the results showed that deeper than 200 m, mean body size decreased and mean trophic level stayed at near 3.70 to 2,300 m depth, and then decreased deeper than 2,300 m. Thus, the depth gradient of mean body size did not follow the temperature-size rule (TSR) but did follow the gill-oxygen limitation theory (GOLT) because the dissolved oxygen (DO) decreased with depth (Costello et al., 2018).

Fewer than 40 species in our dataset occur deeper than 2,300 m, and most of them were high-trophic-level omnivores, with less than 10 of these species being carnivorous (Fig. 6). Without photosynthesis, deep-sea species mostly rely on detritus from the surface waters (known as marine snow), consisting of dead or dying animals and phytoplankton and faecal matter produced by zooplankton, as their primary source of food (Higgs, Gates & Jones, 2014). Only 5% of food can fall into the bathypelagic zone, so bathypelagic fish prey on anything that comes their way (Ryan, 2006). Thus, fish assemblages in the deep sea have a more similar environmental and dietary niche than those in shallower depths.

Although the primary trend for mean body size decreased with depth and mean trophic level stayed stable at 3.7 till 2,300 m, there were some depths where values rose or fell. These depths were at 100, 500, 900, 1,400, 1,800, 2,300, and 3,000 m, respectively (Figs. 5A and 6A), and they fit the points of turnover at 100, 500, 1,400, 2,300, and 3,000 m in species assemblages from clustering analysis in Lin, Costello & Wright (2023). Thus, these changes reflect the modelled depth zonation of fish species assemblages with different ranges of body size and trophic level.

### Environmental relationships

Fish’s body size and trophic level were smaller and lower in the warmer and low DO latitudes (tropics and subtropics) but larger and higher in the cooler and high DO latitudes (temperate areas and the Arctic Ocean but not the Southern Ocean). These results may support hypotheses of temperature-size rule, gill-oxygen limitation theory, and temperature constraint hypotheses. As warmer temperature decreases aerobic capacity, fish with larger body size may be limited by oxygen supply (Pauly, 2010), and thus there are more small fish in the warmer latitudes. Also, body size has a positive relationship with trophic level, so small fish usually have a lower trophic level because of the limitation of gape size (Romanuk, Hayward & Hutchings, 2011), and this relationship is more significant when excluding the lower trophic level fish because some species are large (Keppeler, Montaña & Winemiller, 2020). For most vertebrate ectotherms, the metabolic rate and gut passage rate increase with temperature (Zachariassen, Cossins & Bowler, 1989; Zimmerman & Tracy, 1989; Horn & Gibson, 1990; Van Marken Lichtenbelt, Vogel & Wesselingh, 1997; Gillooly et al., 2001). However, the gut passage rate decreases more rapidly than metabolic rate when temperature declines, so herbivorous fish may not be able to digest enough food material to meet their metabolic demands at cooler temperatures (Floeter et al., 2005). Except for the physical variables, biogenic habitats may also provide niches for more fish species in the tropics, notably coral reefs which harbour about 30% of all fish species (Costello, 2015).

Both the Arctic and Antarctic are polar environments with near freezing temperatures but high DO (Fig. 7) and relatively low species richness compared to other latitudes (Lin et al., 2021). However, we show the Arctic has far more variable environmental conditions than the Antarctic (Fig. 7). The biogeography of their fish fauna also contrasts. The fish fauna in the Arctic Ocean is an extension of that of boreal and temperate regions (Loeng et al., 2005) because of the active northward colonization from the Atlantic and Pacific over the last 6,000–14,000 years (Dayton, Mordida & Bacon, 1994; Eastman, 1997). Of the Arctic fauna, 58% of species comprise six groups of fish, zoarcoids, gadiformes, cottids, salmonids, pleuronectiforms, and chondrichthyans (Eastman, 1997), and only around 20% the fish species are endemic (Eastman, 1997; Reshetnikov, 2004; Mecklenburg, Møller & Steinke, 2011). In contrast, Antarctica is isolated by the Antarctic Circumpolar Current and deep-sea with 88% of fish species (Eastman, 1997; McGonigal & Woodworth, 2003; Duhamel et al., 2014), and over 45% of all its marine species (Costello et al., 2010), being endemic. Five fish groups, namely notothenioids, myctophids, liparids, zoarcids, and gadiforms, accounted for 74% of the Antarctic fish fauna, and notothenioids comprised 35% (Eastman, 1997). Therefore, the Antarctic fish fauna has a closer phylogenetic relationship than that of the Arctic (Lin, Costello & Wright, 2023). The Antarctic also has a simpler food web compared to other latitudes (Richardson, 1975; Targett, 1981; La Mesa, Eastman & Vacchi, 2004; Hill et al., 2006). However, these differences in phylo-biogeography do not necessarily explain the smaller body size and lower trophic level of Antarctic than Arctic fish fauna (Figs. 1–4). Here, we suggest that these differences are due to the greater spatial environmental heterogeneity in the Arctic providing more niches for larger and higher trophic level species than available in the more homogenous seas around Antarctica.

## Conclusions

This study found that mean body sizes and mean trophic levels of marine fish were lower in the tropics and sub-tropics, between 30°S and 30°N, than high latitudes, and less in the deep-sea and Antarctica. The flatter latitudinal gradients of these traits in the deep sea reflect its more homogenous environment. While mean body size generally increases at colder temperatures, the reverse was the case with depth. Body size and trophic level decreased with depth not because of temperature, but because of the low dissolved oxygen and scarce food resources in the deep sea. Thus, the latitudinal gradients at the surface zone support the Temperature-Size Rule, Temperature Constraint hypothesis, and Gill-Oxygen Limitation Theory while the depth gradient only supports the latter. Therefore, our global scale analysis shows that in the surface zone, temperature is the primary and dissolved oxygen is the second factor influencing the biogeography of fish body size and trophic level, whereas oxygen and food supply limit these traits in the deep-sea and Antarctic species.

## Supplemental Information

10.7717/peerj.15880/supp-1 Supplemental Information 1 Supporting Information.

## Additional Information and Declarations

### Data Availability

The following information was supplied regarding data availability: Data is available at Figshare: Lin, Han-Yang; Costello, Mark (2022). Body size and trophic level increase with latitude and decrease in the deep-sea and Antarctica for marine fish species. figshare. Journal contribution. https://doi.org/10.6084/m9.figshare.19314317.v3.
