# Supplementary material for: Body size and trophic level increase with latitude, and decrease in the deep-sea and Antarctica, for marine fish species
Source: PeerJ. 2023 Sep 7;11:e15880. doi: 10.7717/peerj.15880 (PMC10493087; doi:10.7717/peerj.15880)
Supplement: Supplemental Information 1 [file peerj-11-15880-s001.docx]

Supporting Information for

**Body size and trophic level increase with latitude and decrease in the deep-sea and Antarctica for marine fish species**

Table S1. Comparison of species number between FishBase and this study

| **Fish group** | | **FishBase** | | **This Study** | | | |  |  |
| --- | --- | --- | --- | --- | --- | --- | --- | --- | --- |
|  |  | **Species number** | | **Species number** | | **%** | |  |  |
| All fish | | 15195 | | 5619 | | 37 | |  |  |
| With body size records | | 13894 | | 5619 | | 40 | |  |  |
| With trophic level records | | 15194 | | 5619 | | 37 | |  |  |
| **In traits group** |  | |  | |  | |  | |  |
| **Maximum body size (cm)** | **FishBase** | | | | **This Study** | | | | |
|  | **Species number** | | **% of all records** | | **Species number** | | **% of 5619 species** | | **% of FishBase species** |
| < 30 | 8644 | | 62 | | 2743 | | 49 | | 32 |
| 30 - 100 | 4402 | | 32 | | 2260 | | 40 | | 51 |
| > 100 | 848 | | 6 | | 616 | | 11 | | 73 |
| **Trophic level** |  | |  | |  | |  | |  |
| < 2.20 | 468 | | 3 | | 242 | | 4 | | 52 |
| 2.20 - 2.80 | 621 | | 4 | | 286 | | 5 | | 46 |
| 2.81 - 3.70 | 10203 | | 67 | | 3234 | | 58 | | 32 |
| > 3.7 | 3902 | | 26 | | 1857 | | 33 | | 48 |

Table S2. Number of species for groups of three body sizes and four trophic levels in the 5-degree latitude band in the whole water column.

| **Latitude (°)** | **All fish** | **Maximum body size (cm)** | | |  | **Trophic level** | | | |
| --- | --- | --- | --- | --- | --- | --- | --- | --- | --- |
|  |  | **< 30** | **30 - 100** | **> 100** |  | **< 2.20** | **2.21 -2.80** | **2.81 - 3.70** | **> 3.70** |
| **-75** | 72 | 39 | 32 | 1 |  | 0 | 1 | 58 | 13 |
| **-70** | 95 | 52 | 40 | 3 |  | 0 | 1 | 72 | 22 |
| **-65** | 120 | 63 | 52 | 5 |  | 0 | 1 | 94 | 25 |
| **-60** | 177 | 81 | 74 | 22 |  | 2 | 3 | 118 | 54 |
| **-55** | 265 | 95 | 110 | 60 |  | 2 | 4 | 143 | 116 |
| **-50** | 396 | 152 | 151 | 93 |  | 5 | 7 | 209 | 175 |
| **-45** | 581 | 212 | 234 | 135 |  | 10 | 13 | 301 | 257 |
| **-40** | 828 | 298 | 342 | 188 |  | 15 | 25 | 402 | 386 |
| **-35** | 1462 | 550 | 634 | 278 |  | 45 | 66 | 740 | 611 |
| **-30** | 1997 | 845 | 831 | 321 |  | 100 | 111 | 1035 | 751 |
| **-25** | 2543 | 1197 | 988 | 358 |  | 155 | 158 | 1325 | 905 |
| **-20** | 2777 | 1343 | 1063 | 371 |  | 156 | 167 | 1470 | 984 |
| **-15** | 2914 | 1418 | 1119 | 377 |  | 173 | 172 | 1543 | 1026 |
| **-10** | 3090 | 1549 | 1159 | 382 |  | 181 | 188 | 1655 | 1066 |
| **-5** | 3147 | 1572 | 1188 | 387 |  | 183 | 180 | 1690 | 1094 |
| **0** | 3188 | 1590 | 1206 | 392 |  | 187 | 188 | 1706 | 1107 |
| **5** | 3299 | 1661 | 1248 | 390 |  | 192 | 189 | 1781 | 1137 |
| **10** | 3461 | 1743 | 1323 | 395 |  | 196 | 189 | 1894 | 1182 |
| **15** | 3480 | 1722 | 1353 | 405 |  | 197 | 193 | 1895 | 1195 |
| **20** | 3532 | 1721 | 1393 | 418 |  | 196 | 187 | 1946 | 1203 |
| **25** | 3533 | 1664 | 1438 | 431 |  | 180 | 181 | 1940 | 1232 |
| **30** | 3397 | 1551 | 1426 | 420 |  | 171 | 158 | 1865 | 1203 |
| **35** | 2341 | 931 | 1021 | 389 |  | 66 | 81 | 1281 | 913 |
| **40** | 1656 | 610 | 716 | 330 |  | 33 | 40 | 907 | 676 |
| **45** | 1291 | 478 | 552 | 261 |  | 18 | 29 | 726 | 518 |
| **50** | 897 | 325 | 394 | 178 |  | 4 | 17 | 522 | 354 |
| **55** | 750 | 268 | 334 | 148 |  | 1 | 12 | 452 | 285 |
| **60** | 633 | 222 | 277 | 134 |  | 1 | 11 | 367 | 254 |
| **65** | 434 | 147 | 190 | 97 |  | 0 | 5 | 253 | 176 |
| **70** | 216 | 67 | 82 | 67 |  | 0 | 1 | 123 | 92 |
| **75** | 113 | 37 | 43 | 33 |  | 0 | 1 | 66 | 46 |

Table S3. Number of species for groups of three body sizes and four trophic levels in the 5-degree latitude band in the surface zone (0 – 200 m).

| **Latitude (°)** | **All fish** | **Maximum body size (cm)** | | |  | **Trophic level** | | | |
| --- | --- | --- | --- | --- | --- | --- | --- | --- | --- |
|  |  | **< 30** | **30 - 100** | **> 100** |  | **< 2.20** | **2.21 -2.80** | **2.81 - 3.70** | **> 3.70** |
| **-75** | 8 | 4 | 4 | 0 |  | 0 | 0 | 7 | 1 |
| **-70** | 13 | 6 | 7 | 0 |  | 0 | 0 | 9 | 4 |
| **-65** | 16 | 7 | 9 | 0 |  | 0 | 0 | 12 | 4 |
| **-60** | 24 | 11 | 10 | 3 |  | 1 | 2 | 13 | 8 |
| **-55** | 59 | 17 | 29 | 13 |  | 1 | 3 | 27 | 28 |
| **-50** | 112 | 34 | 50 | 28 |  | 4 | 6 | 55 | 47 |
| **-45** | 231 | 72 | 104 | 55 |  | 9 | 12 | 116 | 94 |
| **-40** | 402 | 125 | 181 | 96 |  | 14 | 25 | 186 | 177 |
| **-35** | 908 | 314 | 430 | 164 |  | 44 | 66 | 462 | 336 |
| **-30** | 1406 | 589 | 614 | 203 |  | 99 | 111 | 745 | 451 |
| **-25** | 1931 | 922 | 768 | 241 |  | 154 | 158 | 1032 | 587 |
| **-20** | 2129 | 1042 | 830 | 257 |  | 155 | 167 | 1152 | 655 |
| **-15** | 2264 | 1110 | 883 | 271 |  | 172 | 172 | 1217 | 703 |
| **-10** | 2419 | 1221 | 921 | 277 |  | 180 | 188 | 1313 | 738 |
| **-5** | 2467 | 1237 | 945 | 285 |  | 183 | 180 | 1341 | 763 |
| **0** | 2511 | 1254 | 965 | 292 |  | 187 | 188 | 1357 | 779 |
| **5** | 2595 | 1308 | 995 | 292 |  | 192 | 189 | 1413 | 801 |
| **10** | 2714 | 1376 | 1044 | 294 |  | 196 | 189 | 1502 | 827 |
| **15** | 2715 | 1350 | 1068 | 297 |  | 197 | 192 | 1495 | 831 |
| **20** | 2719 | 1323 | 1095 | 301 |  | 196 | 186 | 1511 | 826 |
| **25** | 2679 | 1263 | 1111 | 305 |  | 180 | 180 | 1484 | 835 |
| **30** | 2517 | 1149 | 1080 | 288 |  | 171 | 156 | 1400 | 790 |
| **35** | 1527 | 576 | 702 | 249 |  | 66 | 80 | 842 | 539 |
| **40** | 952 | 313 | 436 | 203 |  | 33 | 39 | 514 | 366 |
| **45** | 711 | 248 | 318 | 145 |  | 18 | 28 | 398 | 267 |
| **50** | 423 | 151 | 192 | 80 |  | 4 | 16 | 256 | 147 |
| **55** | 345 | 127 | 155 | 63 |  | 1 | 11 | 217 | 116 |
| **60** | 292 | 108 | 132 | 52 |  | 1 | 11 | 179 | 101 |
| **65** | 176 | 63 | 80 | 33 |  | 0 | 5 | 107 | 64 |
| **70** | 98 | 34 | 41 | 23 |  | 0 | 1 | 63 | 34 |
| **75** | 48 | 17 | 21 | 10 |  | 0 | 1 | 30 | 17 |

Table S4. Number of species for groups of three body sizes and four trophic levels in the 5-degree latitude band in the middle zone (201 – 1000 m).

| **Latitude (°)** | **All fish** | **Maximum body size (cm)** | | |  | **Trophic level** | | | |
| --- | --- | --- | --- | --- | --- | --- | --- | --- | --- |
|  |  | **< 30** | **30 - 100** | **> 100** |  | **< 2.20** | **2.21 -2.80** | **2.81 - 3.70** | **> 3.70** |
| **-75** | 50 | 28 | 21 | 1 |  | 0 | 1 | 40 | 9 |
| **-70** | 61 | 36 | 22 | 3 |  | 0 | 1 | 47 | 13 |
| **-65** | 74 | 40 | 30 | 4 |  | 0 | 1 | 59 | 14 |
| **-60** | 97 | 41 | 44 | 12 |  | 1 | 1 | 63 | 32 |
| **-55** | 135 | 40 | 59 | 36 |  | 1 | 1 | 64 | 69 |
| **-50** | 193 | 72 | 70 | 51 |  | 1 | 1 | 92 | 99 |
| **-45** | 246 | 88 | 94 | 64 |  | 1 | 1 | 117 | 127 |
| **-40** | 303 | 115 | 115 | 73 |  | 1 | 0 | 143 | 159 |
| **-35** | 403 | 163 | 146 | 94 |  | 1 | 0 | 189 | 213 |
| **-30** | 441 | 183 | 160 | 98 |  | 1 | 0 | 204 | 236 |
| **-25** | 460 | 203 | 162 | 95 |  | 1 | 0 | 208 | 251 |
| **-20** | 482 | 219 | 171 | 92 |  | 1 | 0 | 224 | 257 |
| **-15** | 487 | 229 | 173 | 85 |  | 1 | 0 | 234 | 252 |
| **-10** | 502 | 246 | 173 | 83 |  | 1 | 0 | 243 | 258 |
| **-5** | 507 | 252 | 173 | 82 |  | 0 | 0 | 245 | 262 |
| **0** | 501 | 254 | 167 | 80 |  | 0 | 0 | 245 | 256 |
| **5** | 525 | 269 | 176 | 80 |  | 0 | 0 | 262 | 263 |
| **10** | 558 | 283 | 193 | 82 |  | 0 | 0 | 281 | 277 |
| **15** | 571 | 288 | 196 | 87 |  | 0 | 1 | 286 | 284 |
| **20** | 606 | 307 | 204 | 95 |  | 0 | 1 | 309 | 296 |
| **25** | 638 | 305 | 231 | 102 |  | 0 | 1 | 325 | 312 |
| **30** | 666 | 308 | 252 | 106 |  | 0 | 2 | 337 | 327 |
| **35** | 607 | 269 | 225 | 113 |  | 0 | 1 | 317 | 289 |
| **40** | 525 | 223 | 198 | 104 |  | 0 | 1 | 284 | 240 |
| **45** | 425 | 171 | 161 | 93 |  | 0 | 1 | 235 | 189 |
| **50** | 340 | 130 | 134 | 76 |  | 0 | 1 | 189 | 150 |
| **55** | 288 | 102 | 122 | 64 |  | 0 | 1 | 166 | 121 |
| **60** | 253 | 82 | 108 | 63 |  | 0 | 0 | 141 | 112 |
| **65** | 187 | 57 | 81 | 49 |  | 0 | 0 | 108 | 79 |
| **70** | 97 | 27 | 34 | 36 |  | 0 | 0 | 53 | 44 |
| **75** | 56 | 15 | 21 | 20 |  | 0 | 0 | 31 | 25 |

Table S5. Number of species for groups of three body sizes and four trophic levels in the 5-degree latitude band in the deep zone (1001 – 6000 m).

| **Latitude (°)** | **All fish** | **Maximum body size (cm)** | | |  | **Trophic level** | | | |
| --- | --- | --- | --- | --- | --- | --- | --- | --- | --- |
|  |  | **< 30** | **30 - 100** | **> 100** |  | **< 2.20** | **2.21 -2.80** | **2.81 - 3.70** | **> 3.70** |
| **-75** | 14 | 7 | 7 | 0 |  | 0 | 0 | 11 | 3 |
| **-70** | 21 | 10 | 11 | 0 |  | 0 | 0 | 16 | 5 |
| **-65** | 30 | 16 | 13 | 1 |  | 0 | 0 | 23 | 7 |
| **-60** | 56 | 29 | 20 | 7 |  | 0 | 0 | 42 | 14 |
| **-55** | 71 | 38 | 22 | 11 |  | 0 | 0 | 52 | 19 |
| **-50** | 91 | 46 | 31 | 14 |  | 0 | 0 | 62 | 29 |
| **-45** | 104 | 52 | 36 | 16 |  | 0 | 0 | 68 | 36 |
| **-40** | 123 | 58 | 46 | 19 |  | 0 | 0 | 73 | 50 |
| **-35** | 151 | 73 | 58 | 20 |  | 0 | 0 | 89 | 62 |
| **-30** | 150 | 73 | 57 | 20 |  | 0 | 0 | 86 | 64 |
| **-25** | 152 | 72 | 58 | 22 |  | 0 | 0 | 85 | 67 |
| **-20** | 166 | 82 | 62 | 22 |  | 0 | 0 | 94 | 72 |
| **-15** | 163 | 79 | 63 | 21 |  | 0 | 0 | 92 | 71 |
| **-10** | 169 | 82 | 65 | 22 |  | 0 | 0 | 99 | 70 |
| **-5** | 173 | 83 | 70 | 20 |  | 0 | 0 | 104 | 69 |
| **0** | 176 | 82 | 74 | 20 |  | 0 | 0 | 104 | 72 |
| **5** | 179 | 84 | 77 | 18 |  | 0 | 0 | 106 | 73 |
| **10** | 189 | 84 | 86 | 19 |  | 0 | 0 | 111 | 78 |
| **15** | 194 | 84 | 89 | 21 |  | 0 | 0 | 114 | 80 |
| **20** | 207 | 91 | 94 | 22 |  | 0 | 0 | 126 | 81 |
| **25** | 216 | 96 | 96 | 24 |  | 0 | 0 | 131 | 85 |
| **30** | 214 | 94 | 94 | 26 |  | 0 | 0 | 128 | 86 |
| **35** | 207 | 86 | 94 | 27 |  | 0 | 0 | 122 | 85 |
| **40** | 179 | 74 | 82 | 23 |  | 0 | 0 | 109 | 70 |
| **45** | 155 | 59 | 73 | 23 |  | 0 | 0 | 93 | 62 |
| **50** | 134 | 44 | 68 | 22 |  | 0 | 0 | 77 | 57 |
| **55** | 117 | 39 | 57 | 21 |  | 0 | 0 | 69 | 48 |
| **60** | 88 | 32 | 37 | 19 |  | 0 | 0 | 47 | 41 |
| **65** | 71 | 27 | 29 | 15 |  | 0 | 0 | 38 | 33 |
| **70** | 21 | 6 | 7 | 8 |  | 0 | 0 | 7 | 14 |
| **75** | 9 | 5 | 1 | 3 |  | 0 | 0 | 5 | 4 |

Table S6. Number of species for all fish, three body sizes and four trophic levels along 100 m depth bands.

| **Depth band (m)** | **All fish** | **Maximum body size (cm)** | | | **Trophic level** | | | | |  |
| --- | --- | --- | --- | --- | --- | --- | --- | --- | --- | --- |
|  |  | **< 30** | **30 -100** | **> 100** | | **< 2.20** | **2.21 -2.80** | **2.81 - 3.70** | **> 3.70** | |
| **0 - 100** | 4224 | 2104 | 1666 | 454 | | 241 | 285 | 2443 | 1255 | |
| **101 - 200** | 1153 | 428 | 525 | 200 | | 3 | 13 | 597 | 540 | |
| **201 - 300** | 904 | 386 | 358 | 160 | | 1 | 4 | 477 | 422 | |
| **301 - 400** | 736 | 344 | 277 | 115 | | 1 | 2 | 392 | 341 | |
| **401 - 500** | 636 | 294 | 233 | 109 | | 1 | 1 | 332 | 302 | |
| **501 - 600** | 517 | 247 | 187 | 83 | | 1 | 1 | 273 | 242 | |
| **601 - 700** | 444 | 219 | 164 | 61 | | 0 | 0 | 253 | 191 | |
| **701 - 800** | 401 | 192 | 155 | 54 | | 0 | 0 | 240 | 161 | |
| **801 - 900** | 360 | 170 | 143 | 47 | | 0 | 0 | 226 | 134 | |
| **901 - 1000** | 278 | 125 | 114 | 39 | | 0 | 0 | 166 | 112 | |
| **1001 - 1100** | 242 | 107 | 99 | 36 | | 0 | 0 | 141 | 101 | |
| **1101 - 1200** | 206 | 87 | 88 | 31 | | 0 | 0 | 115 | 91 | |
| **1201 - 1300** | 163 | 70 | 69 | 24 | | 0 | 0 | 90 | 73 | |
| **1301 - 1400** | 131 | 59 | 54 | 18 | | 0 | 0 | 74 | 57 | |
| **1401 - 1500** | 138 | 75 | 48 | 15 | | 0 | 0 | 79 | 59 | |
| **1501 - 1600** | 129 | 72 | 46 | 11 | | 0 | 0 | 74 | 55 | |
| **1601 - 1700** | 112 | 63 | 41 | 8 | | 0 | 0 | 66 | 46 | |
| **1701 - 1800** | 100 | 54 | 38 | 8 | | 0 | 0 | 56 | 44 | |
| **1801 - 1900** | 92 | 49 | 36 | 7 | | 0 | 0 | 54 | 38 | |
| **1901 - 2000** | 82 | 44 | 33 | 5 | | 0 | 0 | 47 | 35 | |
| **2001 - 2100** | 77 | 43 | 28 | 6 | | 0 | 0 | 47 | 30 | |
| **2101 - 2200** | 63 | 39 | 20 | 4 | | 0 | 0 | 40 | 23 | |
| **2201 - 2300** | 59 | 37 | 18 | 4 | | 0 | 0 | 36 | 23 | |
| **2301 - 2400** | 36 | 17 | 16 | 3 | | 0 | 0 | 26 | 10 | |
| **2401 - 2500** | 32 | 15 | 14 | 3 | | 0 | 0 | 23 | 9 | |
| **2501 - 2600** | 23 | 12 | 9 | 2 | | 0 | 0 | 16 | 7 | |
| **2601 - 2700** | 13 | 6 | 6 | 1 | | 0 | 0 | 10 | 3 | |
| **2701 - 2800** | 14 | 6 | 7 | 1 | | 0 | 0 | 11 | 3 | |
| **2801 - 2900** | 15 | 5 | 9 | 1 | | 0 | 0 | 12 | 3 | |
| **2901 - 3000** | 14 | 4 | 9 | 1 | | 0 | 0 | 11 | 3 | |
| **3001 - 3100** | 14 | 4 | 9 | 1 | | 0 | 0 | 12 | 2 | |
| **3101 - 3200** | 12 | 3 | 9 | 0 | | 0 | 0 | 11 | 1 | |
| **3201 - 3300** | 10 | 2 | 8 | 0 | | 0 | 0 | 9 | 1 | |
| **3301 - 3400** | 8 | 2 | 6 | 0 | | 0 | 0 | 7 | 1 | |
| **3401 - 3500** | 6 | 2 | 4 | 0 | | 0 | 0 | 5 | 1 | |

Table S7. Mean and standard error (± SE) of maximum body size (cm) and trophic level for all fish in 5-degree latitude bands.

|  | **Maximum body size (cm)** | | | | | | | | | **Trophic level** | | | | | | | | | |
| --- | --- | --- | --- | --- | --- | --- | --- | --- | --- | --- | --- | --- | --- | --- | --- | --- | --- | --- | --- |
| **Latitude (°)** | **Whole water column** | | **0 - 200 m** | | **201 - 1000 m** | | **1000 - 6000 m** | | | **Whole water column** | | **0 - 200 m** | | **201 - 1000 m** | | **1000 - 6000 m** | | | |
|  | **Mean** | **Standard error** | **Mean** | **Standard error** | **Mean** | **Standard error** | **Mean** | **Standard error** | **Mean** | | **Standard error** | **Mean** | **Standard error** | **Mean** | **Standard error** | **Mean** | **Standard error** | |  |
| **-75** | 33.6 | 3.2 | 26.5 | 9.4 | 32.6 | 4.1 | 35.2 | 6.2 | 3.4 | | 0.0 | 3.4 | 0.6 | 3.4 | 0.0 | 3.5 | 0.1 |  |  |
| **-70** | 35.5 | 3.1 | 33.4 | 9.3 | 33.0 | 3.9 | 37.6 | 5.9 | 3.5 | | 0.0 | 3.6 | 1.2 | 3.5 | 0.0 | 3.5 | 0.1 |  |  |
| **-65** | 39.7 | 4.0 | 31.8 | 8.0 | 38.9 | 5.4 | 39.9 | 7.6 | 3.5 | | 0.0 | 3.6 | 1.1 | 3.5 | 0.0 | 3.5 | 0.1 |  |  |
| **-60** | 66.4 | 10.4 | 138.4 | 28.3 | 73.3 | 17.3 | 47.9 | 6.9 | 3.6 | | 0.0 | 3.5 | 0.4 | 3.6 | 0.0 | 3.5 | 0.0 |  |  |
| **-55** | 87.5 | 8.6 | 151.4 | 19.7 | 98.2 | 14.0 | 50.0 | 6.4 | 3.7 | | 0.0 | 3.7 | 0.2 | 3.7 | 0.0 | 3.6 | 0.0 |  |  |
| **-50** | 89.9 | 8.2 | 228.9 | 21.6 | 89.9 | 10.6 | 52.2 | 7.1 | 3.7 | | 0.0 | 3.6 | 0.3 | 3.8 | 0.0 | 3.6 | 0.0 |  |  |
| **-45** | 86.0 | 6.0 | 173.5 | 11.4 | 88.4 | 8.8 | 52.1 | 6.4 | 3.7 | | 0.0 | 3.6 | 0.3 | 3.8 | 0.0 | 3.6 | 0.0 |  |  |
| **-40** | 83.2 | 4.6 | 150.5 | 7.5 | 81.3 | 7.3 | 52.5 | 5.6 | 3.7 | | 0.0 | 3.6 | 0.0 | 3.8 | 0.0 | 3.7 | 0.0 |  |  |
| **-35** | 73.8 | 3.0 | 120.6 | 4.0 | 78.9 | 5.8 | 49.5 | 4.7 | 3.6 | | 0.0 | 3.5 | 0.1 | 3.8 | 0.0 | 3.7 | 0.0 |  |  |
| **-30** | 65.1 | 2.3 | 103.4 | 2.8 | 75.0 | 5.4 | 48.7 | 4.6 | 3.5 | | 0.0 | 3.4 | -0.1 | 3.8 | 0.0 | 3.7 | 0.0 |  |  |
| **-25** | 58.8 | 1.9 | 93.0 | 2.1 | 71.6 | 5.2 | 50.3 | 4.7 | 3.5 | | 0.0 | 3.4 | -0.2 | 3.8 | 0.0 | 3.7 | 0.0 |  |  |
| **-20** | 56.8 | 1.8 | 91.0 | 2.0 | 68.7 | 5.0 | 47.9 | 4.3 | 3.5 | | 0.0 | 3.4 | -0.2 | 3.8 | 0.0 | 3.7 | 0.0 |  |  |
| **-15** | 56.2 | 1.7 | 90.2 | 1.9 | 66.6 | 4.9 | 48.0 | 4.4 | 3.5 | | 0.0 | 3.4 | -0.2 | 3.7 | 0.0 | 3.7 | 0.0 |  |  |
| **-10** | 54.7 | 1.6 | 88.2 | 1.8 | 64.9 | 4.8 | 48.2 | 4.3 | 3.5 | | 0.0 | 3.4 | -0.2 | 3.7 | 0.0 | 3.7 | 0.0 |  |  |
| **-5** | 54.7 | 1.6 | 87.8 | 1.8 | 64.2 | 4.7 | 45.8 | 3.9 | 3.5 | | 0.0 | 3.4 | -0.2 | 3.7 | 0.0 | 3.7 | 0.0 |  |  |
| **0** | 54.6 | 1.6 | 87.3 | 1.7 | 63.8 | 4.8 | 46.3 | 3.9 | 3.5 | | 0.0 | 3.4 | -0.2 | 3.7 | 0.0 | 3.7 | 0.0 |  |  |
| **5** | 53.7 | 1.5 | 85.9 | 1.7 | 62.2 | 4.6 | 45.4 | 3.7 | 3.5 | | 0.0 | 3.4 | -0.2 | 3.7 | 0.0 | 3.7 | 0.0 |  |  |
| **10** | 53.0 | 1.5 | 84.5 | 1.6 | 61.4 | 4.3 | 46.5 | 3.6 | 3.5 | | 0.0 | 3.4 | -0.2 | 3.7 | 0.0 | 3.7 | 0.0 |  |  |
| **15** | 53.7 | 1.5 | 85.0 | 1.6 | 62.0 | 4.3 | 47.7 | 3.5 | 3.5 | | 0.0 | 3.4 | -0.2 | 3.7 | 0.0 | 3.7 | 0.0 |  |  |
| **20** | 54.1 | 1.4 | 84.8 | 1.6 | 61.2 | 4.1 | 47.3 | 3.4 | 3.5 | | 0.0 | 3.4 | -0.2 | 3.7 | 0.0 | 3.7 | 0.0 |  |  |
| **25** | 55.1 | 1.4 | 85.1 | 1.6 | 62.3 | 3.9 | 47.6 | 3.3 | 3.5 | | 0.0 | 3.4 | -0.2 | 3.7 | 0.0 | 3.7 | 0.0 |  |  |
| **30** | 56.2 | 1.5 | 86.6 | 1.7 | 62.7 | 3.7 | 48.7 | 3.4 | 3.5 | | 0.0 | 3.4 | -0.2 | 3.7 | 0.0 | 3.7 | 0.0 |  |  |
| **35** | 66.0 | 2.0 | 100.1 | 2.6 | 67.9 | 4.2 | 50.6 | 3.5 | 3.6 | | 0.0 | 3.5 | 0.2 | 3.7 | 0.0 | 3.7 | 0.0 |  |  |
| **40** | 74.8 | 2.7 | 120.1 | 3.9 | 72.0 | 4.8 | 51.5 | 3.9 | 3.6 | | 0.0 | 3.6 | 0.3 | 3.7 | 0.0 | 3.7 | 0.0 |  |  |
| **45** | 76.5 | 3.3 | 128.9 | 4.8 | 77.0 | 5.7 | 55.0 | 4.4 | 3.6 | | 0.0 | 3.6 | 0.4 | 3.7 | 0.0 | 3.7 | 0.0 |  |  |
| **50** | 76.2 | 4.2 | 142.4 | 6.9 | 79.3 | 6.7 | 59.6 | 5.0 | 3.6 | | 0.0 | 3.6 | 0.6 | 3.7 | 0.0 | 3.7 | 0.0 |  |  |
| **55** | 76.8 | 4.8 | 152.2 | 8.2 | 79.5 | 7.4 | 61.1 | 5.5 | 3.6 | | 0.0 | 3.6 | 0.6 | 3.7 | 0.0 | 3.7 | 0.0 |  |  |
| **60** | 75.9 | 4.5 | 104.6 | 6.1 | 84.7 | 8.3 | 64.5 | 7.0 | 3.7 | | 0.0 | 3.6 | 0.6 | 3.7 | 0.0 | 3.7 | 0.0 |  |  |
| **65** | 82.0 | 6.0 | 113.2 | 8.5 | 94.4 | 10.9 | 64.0 | 8.2 | 3.7 | | 0.0 | 3.6 | 0.7 | 3.7 | 0.0 | 3.7 | 0.1 |  |  |
| **70** | 98.6 | 10.4 | 123.7 | 12.5 | 117.8 | 19.1 | 79.8 | 13.5 | 3.7 | | 0.0 | 3.7 | 1.0 | 3.7 | 0.0 | 3.9 | 0.1 |  |  |
| **75** | 97.2 | 16.4 | 90.1 | 13.0 | 128.7 | 30.6 | 57.6 | 23.0 | 3.7 | | 0.0 | 3.7 | 0.7 | 3.7 | 0.1 | 3.9 | 0.2 |  |  |

Table S8. Mean and standard error (± SE) of maximum body size (cm) and trophic level for all fish along 100 m depth bands

| **Depth band (m)** | **Maximum body size (cm)** | | **Trophic level** | |
| --- | --- | --- | --- | --- |
|  | **Mean** | **SE** | **Mean** | **SE** |
| **0 - 100** | 50.6 | 1.2 | 3.4 | 0.0 |
| **101 - 200** | 69.0 | 2.8 | 3.7 | 0.0 |
| **201 - 300** | 66.9 | 3.3 | 3.7 | 0.0 |
| **301 - 400** | 60.0 | 3.5 | 3.7 | 0.0 |
| **401 - 500** | 62.0 | 3.9 | 3.7 | 0.0 |
| **501 - 600** | 59.6 | 4.4 | 3.7 | 0.0 |
| **601 - 700** | 55.7 | 4.8 | 3.7 | 0.0 |
| **701 - 800** | 55.0 | 4.9 | 3.7 | 0.0 |
| **801 - 900** | 55.6 | 5.4 | 3.7 | 0.0 |
| **901 - 1000** | 52.8 | 3.8 | 3.7 | 0.0 |
| **1001 - 1100** | 50.7 | 3.4 | 3.7 | 0.0 |
| **1101 - 1200** | 52.1 | 3.8 | 3.7 | 0.0 |
| **1201 - 1300** | 48.8 | 3.4 | 3.7 | 0.0 |
| **1301 - 1400** | 46.8 | 3.6 | 3.7 | 0.0 |
| **1401 - 1500** | 42.1 | 3.3 | 3.7 | 0.0 |
| **1501 - 1600** | 40.7 | 3.4 | 3.7 | 0.0 |
| **1601 - 1700** | 39.3 | 3.4 | 3.7 | 0.0 |
| **1701 - 1800** | 40.7 | 3.6 | 3.7 | 0.0 |
| **1801 - 1900** | 41.1 | 3.8 | 3.7 | 0.0 |
| **1901 - 2000** | 39.3 | 3.5 | 3.7 | 0.1 |
| **2001 - 2100** | 40.9 | 3.9 | 3.7 | 0.1 |
| **2101 - 2200** | 35.2 | 3.9 | 3.6 | 0.1 |
| **2201 - 2300** | 35.2 | 4.2 | 3.7 | 0.1 |
| **2301 - 2400** | 42.2 | 5.7 | 3.5 | 0.1 |
| **2401 - 2500** | 39.9 | 6.1 | 3.5 | 0.1 |
| **2501 - 2600** | 40.1 | 7.0 | 3.6 | 0.1 |
| **2601 - 2700** | 40.8 | 9.6 | 3.5 | 0.1 |
| **2701 - 2800** | 43.4 | 9.0 | 3.5 | 0.1 |
| **2801 - 2900** | 45.3 | 8.0 | 3.5 | 0.1 |
| **2901 - 3000** | 48.0 | 8.1 | 3.5 | 0.1 |
| **3001 - 3100** | 49.5 | 8.1 | 3.5 | 0.1 |
| **3101 - 3200** | 42.8 | 5.0 | 3.4 | 0.1 |
| **3201 - 3300** | 43.9 | 4.9 | 3.4 | 0.1 |
| **3301 - 3400** | 41.2 | 4.6 | 3.4 | 0.1 |
| **3401 - 3500** | 39.6 | 6.0 | 3.3 | 0.1 |


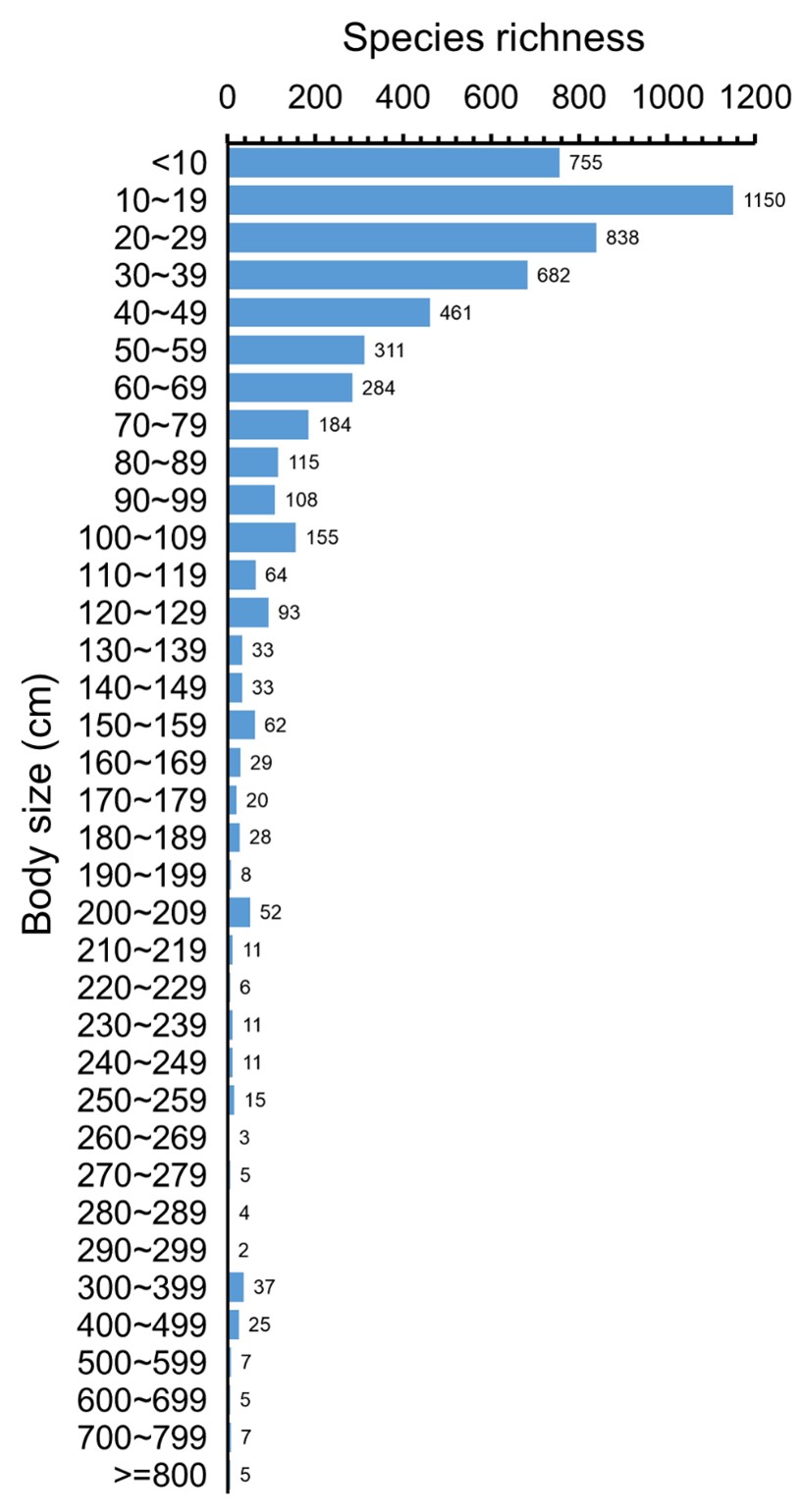


Figure S1. Number of species of marine fish in different body size groups.


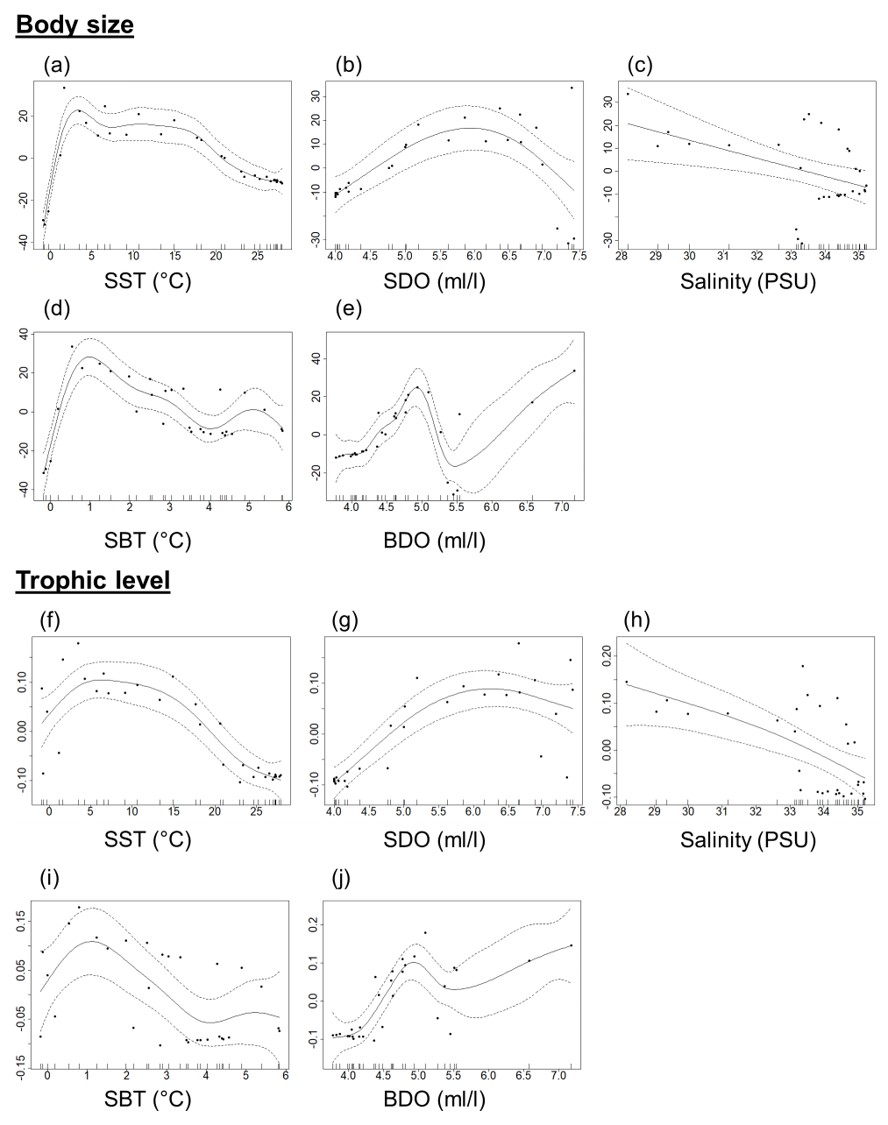


Figure S2: The smoothed GAM (solid lines) for (a – e) mean body size and (f – j) mean trophic level of all fish against sea surface temperature (SST), dissolved oxygen (SDO), salinity, and sea bottom temperature (SBT) and dissolved oxygen (BDO) in 5-degree latitude bands. The dashed lines indicate the 95% confidence intervals. The black dots and tick marks on the x-axis are observed data points. The y-axis represents the spline function. PSU is practical salinity units.
